# Gambling-related suicide in Victoria, Australia: a population-based cross-sectional study

**DOI:** 10.1016/j.lanwpc.2023.100903

**Published:** 2023-09-12

**Authors:** Angela Rintoul, Jeremy Dwyer, Ciara Millar, Lyndal Bugeja, Huy Nguyen

**Affiliations:** a Health Innovation and Transformation Centre (HITC), Federation University, Building 5N, Churchill, Victoria 3842, Australia; b The Department of Forensic Medicine, School of Public Health and Preventive Medicine, Monash University, 65 Kavanagh St, Southbank, Victoria 3006, Australia; c Coroners Prevention Unit, Coroners Court of Victoria, 65 Kavanagh St, Southbank, Victoria 3006, Australia; d HITC, Federation University, Buidling Y, University Drive, Mount Helen, 3350, Victoria, Australia; e Department of Population and Quantitative Health Sciences, University of Massachusetts Medical School, Worcester, USA

**Keywords:** Gambling, Suicide prevention, Suicide epidemiology, Commercial determinants of health, Harm reduction, Gambling treatment

## Abstract

**Background:**

Gambling is associated with serious harms to health, including suicide. Yet public health systems for recording the role of gambling in suicide deaths are relatively underdeveloped. This study contributes to the understanding of this relationship.

**Methods:**

A population-based cross-sectional study of suicides reported to the Coroners Court of Victoria between 2009 and 2016 was performed to identify the incidence and characteristics of gambling-related suicides (GRS).

**Findings:**

From 2009 to 2016 there were 4788 suicide deaths in Victoria. Of these, 184 were identified as direct GRS and a further 17 were GRS by ‘affected others’. Together, these GRS comprise 4.2% of all suicides in Victoria over this eight-year period. Direct GRS account for an annual average rate of 5.13 GRS per million Victorian adults. GRS were significantly more likely to be male (n = 153, 83%), than the Victorian population of total suicide deaths and significantly more likely to occur among those most disadvantaged. Family members and friends were more likely than clinicians to know about the deceased gambling.

**Interpretation:**

Given that gambling is not routinely investigated by coroners and may be hidden from family, friends, and health professionals, this is an underestimate of the true scale of the GRS in Victoria. A range of measures should be introduced to prevent, screen, support, and treat gambling harm. Family members and friends should also be provided with help services. Preventing gambling-related harm through public health measures could significantly reduce suicidality and suicide, both in Australia and globally.

**Funding:**

Federation University Australia, Coroners Court of Victoria, Suicide Prevention Australia.

**Research in context**

**Evidence before this study**

People who gamble at high-risk levels are known to experience suicidality and suicide at higher levels than the general population. Previous studies have demonstrated that electronic gambling machines (EGMs) are most commonly associated with harm and suicide. It has been 25 years since a case series was published using coronial data from Victoria, when systems for recording suicide were less well developed, and when gambling products were not as widely promoted. Few studies have described the incidence or epidemiology of gambling-related suicide (GRS) using coronial datasets. We searched Medline and Web of Science for studies published between 1998 and 2023 to understand current evidence of the magnitude, characteristics, and mechanisms of GRS.

**Added value of this study**

We reviewed the Victorian Suicide Register (VSR) to identify cases of GRS. VSR data is drawn from coroners’ investigations. There are many reasons why the investigation may not uncover evidence of a gambling context, including the deceased concealing their gambling from loved ones, or loved ones being unwilling to disclose it to the coroner because of shame and stigma. Even given these limitations, we identified 184 cases of direct GRS, and 17 ‘affected others’ which account for at least 4.2% of all suicides in Victoria during 2009–2016. While this is almost certainly an undercount of the true rate of cases, it represents a considerable proportion of all suicide cases. Gambling was rarely the only stressor the deceased was experiencing proximal to the fatal incident, with mental ill health, substance use, conflict with partner and work-related stressors, particularly prevalent. These were observed to intersect with and exacerbate one another, highlighting that treatment of gambling problems may need to be delivered as part of a more holistic health and wellbeing program. To date there has been an emphasis on training clinicians to screen for gambling problems in primary care. While this is important, our study found that friends and family were more likely to have knowledge of the deceased gambling problems than their treating health professionals. This indicates that public education to inform family members and friends about support services for themselves as well as those who gamble may be more likely to prevent GRS in the short term.

**Implications of all the available evidence**

This study shows that gambling contributes to a considerable proportion of suicide cases. Coroners could consider whether there may be opportunities in the death investigation process to improve the collection of information about gambling contexts in suicides. Health and help professionals, should also be alert to the potential for gambling to be a contributing or causal factor in suicide and consider the ways in which it may exacerbate other known suicide risks such as relationship breakdown, substance use and mental illnesses. Family members and friends of people who gamble at high-risk levels should be provided with information and assistance to access help. Finally, regulatory measures based on public health approaches should be implemented urgently. This should include the introduction of universal, binding pre-commitment systems, ending gambling advertising, and other upstream preventive measures.

## Introduction

Gambling is associated with a range of harms to health such as family violence^1^^,^^2^ social isolation, neglect of children, obesity, substance use, insomnia, and mental illnesses such as depression and anxiety.^3^, ^4^, ^5^ In 2016 the social cost of gambling in the Australian state of Victoria was estimated to be around $AU7 billion per year.^6^ This is significantly less than the revenue raised by the state government through gambling taxation, which in 2016 was $AU1.7bn.^f^ Harms are experienced not only by the person gambling but by ‘affected others’.^7^ For the person who gambles at high-risk levels it is estimated around six others are directly affected by their gambling.^8^ These include partners, children, parents, siblings, friends, and colleagues. Electronic gambling machines (EGMs) are the product most associated with harmful gambling; this product alone is responsible for over half of all gambling problems in Australia.^9^

The commercial determinants of health have been described as ‘the systems, practices, and pathways through which commercial actors drive health and equity’.^10^ In the context of gambling, one way gambling economic operators shape health is by framing gambling as a recreational pursuit, with the dominant ‘responsible gambling’ paradigm locating responsibility for harm with the individual. This careful framing deflects attention from the regulatory and commercial systems and practices that create the conditions under which gambling is consumed.^11^^,^^12^ These conditions have been conceptualised as commercial determinants of suicide.^13^

This reliance on a flawed paradigm can readily lead to stigma and shame, common among people who gamble at high-risk levels.^14^ As a result, people may be reluctant to seek help for their gambling problems or disclose the nature of their distress to treating health professionals or family members. This framing has been shown to contribute to self-blame and feelings of worthlessness which in turn may contribute to suicidality or suicide.^15^

Gambling disorder (GD) is recognised as a behavioural addiction, classified alongside substance use disorders in the DSM-V and ICD-11. A geographically and methodologically diverse evidence base has emerged showing gambling is associated with excess mortality, including suicidality and suicide. While multiple stressors may be present at the time of death, scoping reviews have found a direct link between gambling and suicide.^16^ A Swedish registry study found that people with diagnosed GD were at 1.8 times higher risk of all-cause mortality than the general population, and 15 times more likely to die by suicide than those without GD.^17^ A study using seven years of financial transaction data from 10% of the United Kingdom (UK) population found that gambling at its highest levels was associated with significantly higher rates of mortality.^18^ An online panel survey of young adults in the UK found a higher adjusted odds ratio of suicidality among young people (4.1–19.7] OR 9 for men and OR 4.5 (2.0–12.0) for women with problem gambling severity index (PGSI) of 8+, compared to those with a PGSI of zero.^19^ A study from Germany found among those gambling at high levels, EGMs increased the risk of suicide events by an odds ratio of 2.85, after controlling for co-morbidities such as mood and personality disorders.^20^ A spatial study of gambling venues and suicide mortality found that the density of gambling venues increases suicide mortality in the United States.^21^ A systematic review of qualitative GRS evidence found the mechanism related to financial distress and shame.^15^

There are few studies of GRS available globally, most published evidence has focussed on gambling-related suicidal ideation, attempts, and self-harm.^16^ A scoping review found six published studies using coronial documents to investigate gambling-related suicides over 20 years to 2016.^16^ This included one study from Victoria, Australia that reviewed a series of 44 coroner-identified cases of gambling suicide from 1990 to 1997.^22^ A study of coronial documents in Hong Kong found that around 19.4% of all suicides were gambling-related, of these, 46% had evidence of financial debts.^23^ Media have reported 18 gambling-related suicides in Kenya, Uganda and Tanzania between January 2014 and June 2021.^24^ Journalists in Quebec, Canada identified 400 cases of GRS in coronial records in the two decades to 2018.^25^ The objective of the current paper is to examine the magnitude and characteristics of GRS in Victoria, Australia during the period 2009–2016, to better understand the context within which the suicides occur so that appropriate responses can be identified.

## Methods

This study was designed, performed and reported in accordance with the Strengthening the Reporting of Observational studies in Epidemiology (STROBE) checklist for cross-sectional studies.

### Study design

The research design comprised a retrospective cross-sectional study of suicides where there was evidence that gambling contributed to death. The study period was eight years (1 January 2009 and 31 December 2016) and the data collection period was five years (November 2016 and November 2021).

### Setting

The study setting was the south-eastern state of Victoria, Australia which had a population of 5,371,934^g^ in 2009 and 6,173,172 in 2016. In Victoria, deaths where suicide is suspected are legally required to be reported to the Coroners Court of Victoria for investigation in accordance with the *Coroners Act 2008* (Vic).

### Participants

Cases were included in the study where the coroner had completed an investigation into the death, the manner of death was determined as suicide, case information had been entered into the Victorian Suicide Register (VSR), and the research team classified the death as related to harm from gambling. Free text information entered in the VSR for the variable “financial stressor” was reviewed by two members of the research team (AR, CM) to identify evidence relating to gambling. A search of terms related to gambling products, location, and providers was also undertaken to account for the potential the deceased was experiencing harm that is not related to financial distress. These terms included gambling, gaming, wagering, betting, racing, casino, EGMs, electronic gam∗machine, pokies, poker machines, table games, roulette, blackjack, baccarat, sic bo, TAB, Sportsbet. Each case was classified as either: confirmed gambling-related; possibly gambling-related; affected other; not gambling-related. To mitigate potential coder bias a third member of the research team (JD) reviewed all confirmed GRS and possible GRS cases to determine agreement.

Cases were classified as “gambling-related” if gambling was identified in the evidence as a contributing factor in the decision to suicide, for example by the deceased in a suicide note or letter, by a witness, the police, and/or the coroner. Cases were classified as “possibly gambling-related” if there was evidence of gambling proximal to death but no direct evidence of a link between gambling harms and the decision to suicide. Finally, cases were classified as “not gambling-related” if there was no evidence of gambling. Examples are provided in Table 1. A cautious approach was taken, such that if for example the deceased attended a gambling venue proximate to death but there was no specific mention of the deceased having gambled this would be “not gambling-related”. Finally, cases were classified as “affected others” if there was evidence the deceased was significantly affected by the gambling of someone close to them proximal to suicide. Affected others are not analysed in this paper.

**Table 1:** Case examples of probable and possible gambling-related suicides.

**Gambling-related suicide case example extracts**
**Case example 1:** Simon^a^ developed a gambling problem which led to significant personal and family distress during the last two years of his life. A year prior to his death, Simon and his family received notice to vacate a house … due to unpaid rent. However, Simon borrowed money from his parents to pay the overdue rent, which meant the family could remain in the house. Simon’s wife discovered her husband had made numerous large withdrawals at a poker machine venue.
**Case example 2:** Gavin^a^ had a gambling addiction and spent a lot of time at the casino. He placed significantly large bets over a six-month period. Police located suicide notes in his bedroom. These notes indicated that Gavin owed significant amounts of money to multiple acquaintances. The notes also spoke of Gavin’s addiction to gambling and indicated his regret at having disappointed his parents.
**Case example 3:** Hùng^a^ had an addiction to gambling, which led to financial stressors. He owed a substantial tax debt and was receiving correspondence from collection agencies for other overdue bills. His bank account records showed regular transfers to an online casino. He sought and received financial assistance from his parents. His medical history included addiction problems, centred on gambling, illicit substances, and alcohol.
**Case example 4:** Darren^a^ attended his doctor and reported feeling depressed in relation to gambling and stated that he becomes angry easily feeling that his life is worthless and hopeless. In the months leading up to his death, Darren attended a gaming venue daily to play poker machines and place bets at the TAB. Darren's best friend saw him just before his death and suspected that Darren was depressed. Darren told him that he didn’t have enough money for food.
**Possibly gambling-related suicide case example extract**
**Case example 1:** Belinda^a^ told friends that if she was ever going to commit suicide that she would do it at a gambling venue. Her treating doctor noted that Belinda had recently been struggling with significant financial concerns and issues with her partner. Her house deposit savings were being steadily eroded and Belinda was concerned about where she and her partner would live.
**Case example 2:** For several months leading up to her death, Veronica^a^ would leave her children at home to attend a gambling venue all night, then come home drunk the next morning, Veronica’s mother was aware she used amphetamines, but did not believe she had any issue with addiction.
**Case Example 3:** Some time ago Alan^a^ spent a week in the Casino with his partner, and she stated that he taught himself to count cards. Alan also enjoyed gambling on horse races. He often told people that he wanted to go to the Casino and gamble a large amount of money.

a Pseudonyms have been used to de-identify the deceased. Cases have been edited lightly for anonymity.

The extracts below show de-identified evidence from cases classified as possible or probably related to their own gambling.

A detailed outline of study variables is provided in Supplementary Materials.

### Data sources and measurement

The primary data source was the VSR, a database of suicides occurring in Victoria that is maintained by the Coroners Court of Victoria. Cases are entered into the VSR by trained coders who review all the material derived from the coroner’s investigation. VSR coding is completed in accordance with a coding manual and data dictionary. The coronial material reviewed for VSR coding in a death usually comprises the report of death to the coroner completed by the police or medical professional; post-mortem medical and scientific reports (autopsy, toxicology, and other scientific reports directed by the coroner), a brief of evidence (comprising statements from health and other relevant service providers, family, friends, and witnesses to the incident, as directed by the coroner) and the coroner’s finding. The VSR does not have a dedicated field to capture gambling activity. Instead, coders are instructed to record any evidence about gambling as free text in the ‘contextual stressors’ VSR field and check the ‘financial stressors’ flag.

The usual residential address of the deceased was matched to the 2011 Australian Bureau of Statistics (ABS) statistical area 1 (SA1) rankings of the Victorian index of relative socioeconomic disadvantage (IRSD). This ranking consists of the smallest geographical unit and is a summary measure of a range of indicators of disadvantage. For instance, an area with a low score is likely to have many people with low levels of education, employed in low-skilled jobs, or many households with low income. IRSD deciles were converted into quintiles, from quintile 1 (most disadvantaged) to quintile 5 (least disadvantaged). The residential address of each deceased case was allocated to a rural or metropolitan area according to ABS classification and compared to the distribution of the entire Victorian population.

### Statistical methods

Data was extracted from the VSR into Stata 16.1 for analysis. *Z*-statistic was computed to compare one-sample proportion of GRS for males and females against all suicides in Victoria over this same time period. The proportion of those employed and not in a relationship was compared between males and females with a χ^2^ test. A one-sample Pearson χ^2^ statistic was also used to evaluate whether the GRS were uniformly distributed across Victorian IRSD quintiles. GRS incidence rate ratios and CIs were calculated for each quintile using Poisson regression. A one-sample test of proportions using *Z*-statistic was performed to test the significance of whether those born overseas reflected the proportion of the Victoria population born overseas. This test was also used to assess if the usual residential address of descendants reflected the proportion of the Victorian metropolitan and rural population. A sensitivity analysis was not performed as the entire population of GRS deaths was included. Summary characteristics of the deceased are presented in Table 2, Table 3, Table 4, Table 5.

**Table 2:** Sociodemographic characteristics.

Characteristic	Male n (%)	Female n (%)	Total N (%)	*p* value
Sex	153 (83.2)	31 (16.9)	184 (100)	0.010^a^
Age (y)				
Mean age	43.2	47.9	44	0.088
Age in categories				
17–24	13 (3.9)	1 (7.8)	14 (7.6)	
25–34	37 (24.2)	3 (9.7)	40 (21.7)	
35–44	34 (22.2)	8 (25.8)	42 (22.8)	
45–54	35 (22.9)	9 (29.0)	44 (23.9)	
55–64	24 (15.7)	8 (25.8)	32 (17.4)	
65+	10 (6.5)	2 (6.5)	12 (6.5)	
Relationship status				
Not in a relationship	100 (65.4)	16 (51.6)	116 (63.0)	0.148^b^
Married/de facto/domestic partner	31 (20.2)	12 (38.7)	43 (23.4)	
Dating and other	22 (14.4)	3 (9.7)	23 (13.6)	
Evidence born overseas	47 (30.7)	12 (38.7)	59 (32.1)	0.257^c^^,^^d^
Employment status				
Employed	80 (52.3)	11 (35.5)	91 (49.5)	0.088^b^
Unemployed	36 (23.5)	14 (46.2)	50 (27.2)	
Retired/pensioner	12 (7.8)	2 (6.5)	14 (7.6)	
Unable to work	12 (7.8)	2 (6.5)	14 (7.6)	
Other	1 (0.7)	7 (9.8)	8 (4.4)	
Unknown	7 (4.6)	0 (0)	7 (3.8)	

a Comparison with % of all Victorian suicide cases, derived from the Victorian Suicide Register 2009–2016.

b Comparison of % in a relationship versus all other relationship types.

c Comparison with % of Victorian population born overseas, calculated from: Discover Victoria’s Diverse Population https://www.vic.gov.au/discover-victorias-diverse-population [last accessed 8 May 2023].

d Comparison of % employed versus all other employment status types.

**Table 3:** Socioeconomic status and rural/metropolitan distribution of gambling-related suicide cases, Victoria 2009–2016.

	Total GRS n (%)	Rate/100,000	Rate ratio (95% CI)	Victorian population^b^ N (%) in thousands
Index of relative socioeconomic disadvantage				
Quintile 1 (most disadvantaged)	45 (25.0)	4.32	2.5 (1.4–4.2)	1042 (19.5)
Quintile 2	45 (25.0)	4.28	2.4 (1.4–4.2)	1052 (19.7)
Quintile 3	40 (22.2)	3.73	2.1 (1.2–3.7)	1072 (20.1)
Quintile 4	19 (10.6)	1.76	1 (ref)	1078 (20.2)
Quintile 5 (least disadvantaged)	31 (17.2)	2.85	1.6 (0.9–2.9)	1087 (20.4)
Total^a^	180 (100)	3.38	–	5331 (100)
Location				
Metropolitan	139 (76.4)	3.3	1.1 (0.8–1.5)	4169 (75.3)
Rural (non-metropolitan)	43 (23.6)	3.1	1 (ref)	1369 (24.7)
Total	182 (100)	3.3	–	5537 (100)^c^

a Unallocated/missing SEIFA data for 4 cases, and metro/rural for 2 cases have been excluded so the total will not equal 184 (as in Table 2).

b Victorian population allocated to statistical area level 1, calculated from ABS (2013) Table 3, 2033.0.55.001, SA1 SEIFA Index of Relative Socioeconomic Disadvantage, 2011 [online] https://www.abs.gov.au/ausstats/abs@.nsf/DetailsPage/2033.0.55.0012011?OpenDocument [last accessed 11 July 2023].

c Total Victorian population for SEIFA IRSD and rural/metropolitan area differ as not all residents are allocated a SEIFA score.

**Table 4:** Known gambling information and characteristics.

	Male n (%)	Female n (%)	Total N (%)
Diagnosed gambling disorder	6 (3.9)	2 (6.5)	8 (4.4)
Gambling problem was known by			
Family/friends/colleagues	114 (74.5)	21 (67.8)	135 (73.4)
Clinician	27 (17.7)	11 (35.5)	38 (20.7)
Health professional treated gambling	9 (5.9)	3 (6.7)	12 (6.5)
Most problematic gambling product			
Electronic gambling machines (EGMs)	44 (38.7)	12 (28.8)	56 (30.4)
Wagering (sports betting, racing, both land-based and/or online)	23 (15.1)	0 (0)	23 (12.5)
Casino table games	8 (5.2)	0 (0)	8 (4.4)
Not stated	57 (37.3)	12 (28.8)	69 (37.5)

**Table 5:** Stressors and health conditions.

Stressors	Male n (%)	Female n (%)	Total N (%)
Partner separation/conflict	105 (68.6)	16 (51.6)	121 (65.8)
Substance use	102 (66.7)	16 (51.6)	118 (64.1)
Diagnosed mental illness	74 (48.4)	20 (64.5)	94 (51.1)
Work	77 (50.3)	14 (45.2)	91 (49.5)
Legal	59 (38.6)	8 (25.8)	67 (36.4)
Partner or family violence	36 (23.5)	4 (12.9)	40 (21.7)
Suicide note mentioned gambling	9 (5.9)	3 (9.7)	12 (6.5)
Evidence of impacts			
Finances	114 (74.5)	20 (64.5)	134 (72.8)
Relationships	67 (43.8)	12 (38.7)	79 (42.9)
Housing	22 (14.4)	6 (19.4)	28 (15.2)
Job loss	12 (7.8)	5 (16.3)	17 (9.2)
Legal–criminal/civil	14 (9.2)	3 (9.7)	17 (9.2)

Approval for this study was granted by the Department of Justice Human Research Ethics Committee (Project Number: CF/16/17063) with endorsement from the Victorian State Coroner to access the VSR.

### Role of the funding source

The study funders had no involvement in the study design, data collection, analysis, writing, or in the decision to submit the manuscript for publication.

## Results

**Fig. 1:**
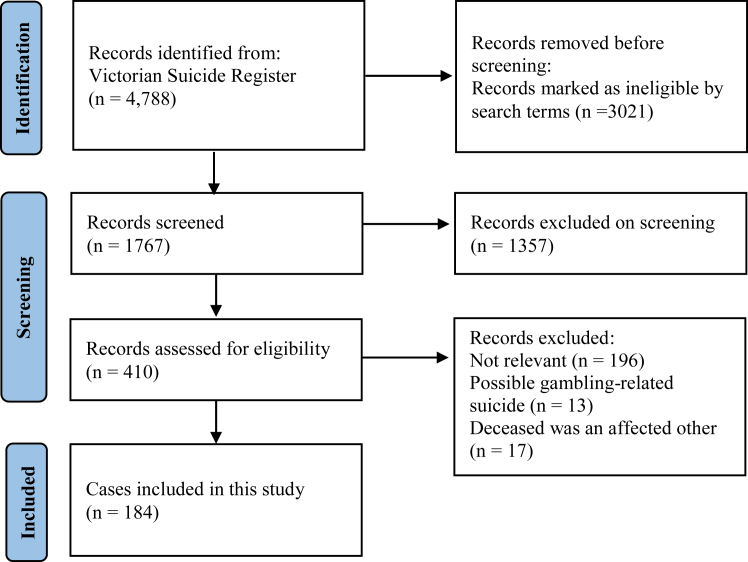
**Identification of cases in the Victorian Suicide Register2009–2016.**

During the eight-year review of VSR records, there were 4788 deaths determined as suicide in Victoria. Our search of the VSR returned 1767 cases for review. Of these, 184 deaths were classified as direct GRS, and a further 17 suicide deaths were ‘affected others’. Together these account for 4.2% of all suicides in Victoria during this time period. There were a further 13 cases that were classified as possibly GRS. The 17 affected other cases are not described further in this paper, which focuses on the 184 GRS cases where the deceased was the person who gambled at harmful levels (Fig. 1).

We found an annual average population rate of 5.13 GRS per million Victorian adults over the 8-year study period. The lowest rate of GRS occurred in 2013 with 3.3 GRS per million Victorian adults and the highest rate was in 2015 with 6.8 per million Victorian adults.

### Descriptive data

A higher proportion of GRS cases were male (83.2%, n = 153, *p* = 0.010) than the overall proportion of suicide cases in Victoria during the same time (75%, n = 3592). Over two-thirds of the males (69.3%, n = 106) were aged 25–54 years while 80.6% (n = 25) of females were aged 35–64 years (*p* = 0.205). Almost two-thirds of males (65.4%, n = 100, *p* = 0.148) were not in a relationship at the time of their death, compared to just over half of females (51.6%, n = 16). Approximately a third of GRS were born overseas (32.1%, n = 59, *p* = 0.257), compared to 28.3% of the 2016 Victorian population born overseas in 2016.^h^ A higher proportion of males (52.3%, n = 80, *p* = 0.088) than females (35.5%, n = 11) were employed while approximately a quarter (23.5%, n = 36) of the males were unemployed and half of the females were unemployed (46.2%, n = 14). Nine deaths occurred among people who had been employed in the gambling industry.

Table 3 shows the SEIFA IRSD socioeconomic indicator and rural/metropolitan location of the deceased. The result of the Poisson regression to estimate rate ratio was presented in the rate ratio column where quintile 4 was treated as the reference category as this quintile had the lowest rate of GRS. GRS does not occur uniformly across the five quintiles of socioeconomic disadvantage (*p* = 0.008). Half of those GRS allocated to a SEIFA IRSD area occurred in quintiles 1 and 2, the most disadvantaged areas, while only 39.3% of the Victorian population live in these lowest quintile areas. While 24.7% of the Victorian population resides outside Melbourne, a slightly lower percentage of GRS deaths (23.6%) occurred in these areas (*p* = 0.737).

Form(s) of gambling that were most problematic for the deceased were often not mentioned in the VSR, meaning there was insufficient data to analyse these characteristics in detail. However, where products were mentioned, the most commonly described forms were EGMs (n = 56, 30.4%), followed by wagering (sports betting and animal racing, land-based and/or online) (n = 23, 12.5%) and casino table games (n = 8, 4.4%). In only 38 cases (20.7%) a clinician (GP, mental health professional, or gambling help service) knew about the deceased’s gambling problems. By contrast, in almost three-quarters of GRS (n = 135, 73.4%) there was evidence that family members or friends had knowledge of the gambling problem. There were only eight cases who had been formally diagnosed with a gambling disorder (4.4%).

Reflecting the results of relationship status in Table 2, there was evidence that over two-thirds of males experienced a relationship breakdown or conflict (68.6%, n = 105) compared with just over half of females (51.6%, n = 16). Similar proportions of males (66.7%, n = 102) and females (51.6%, n = 16) showed evidence of substance use. Almost two-thirds of females had a diagnosed mental illness (64.5%, n = 20) compared with just under half of the males (48.4%, n = 74). In only 12 cases (6.5%), the deceased left a suicide note that referred to the harm they had experienced from gambling. There was evidence in 172 GRS cases (93.5%) that the deceased had at least one other risk factor for suicide, such as a diagnosed mental health condition, relationship, work, legal, family violence, and/or substance use stressor present at the time of their death.

### Limitations

The data for this study was drawn from the VSR, which in turn was manually coded with reference to the material gathered during Victorian coroners’ investigations into suspected suicide deaths. The coroner is reliant primarily on the statements of witnesses (family, friends, colleagues, medical practitioners and similar) for insight into the context of these deaths including relevant stressors. There are several reasons why a witness might be aware of the deceased’s gambling but not disclose it during the investigation: for example, because they may not realise its relevance to the investigation, or because of stigma associated with gambling. A further issue is that even when gambling is reported by witnesses, recorded in the coronial material, and coded in the VSR, there may not be sufficient information to determine whether the gambling met the study definition of harmful gambling. The harms related to gambling can be explicitly described in the evidence (for example diversion of money from essential expenses such as food and rent, or a partner describing a separation due to gambling), but gambling can also cause harms that manifest in ways that may not be directly observed, such as the loss of control or agency. For these reasons, the numbers reported here are likely to be an undercount of the true number of GRS. Further, while gambling may be described as a stressor experienced by the deceased, disentangling the temporal order of this relative to other stressors may be difficult in the absence of other evidence, such as a suicide note. For this reason, we use the term gambling-*related* suicide, as distinct from gambling-*caused* suicide, in this study. There is a time lag in cases that are coded to the VSR, and 2016 is the most recent complete year of cases available for review. The cases reviewed in this study preceded the COVID-19 pandemic, where other research has reported an increase in online gambling among some groups. A widespread cost-of-living crisis has also developed since these cases were reviewed. It is plausible that these additional stressors may lead to an increase GRS. A forensic pathologist ascertained the deceased sex for all cases. Information about gender identity is also recorded in the VSR where available, but it is not recorded systematically for every case. Therefore gender is not reported in this study.

## Discussion

The annual rate of direct GRS per million Victorian adults ranged from 3.33 (2013) to 6.8 (2015). A greater proportion of male GRS were younger, and not in a relationship compared to females. GRS were also more likely to be male than the overall population of GRS in Victoria. While quintile 4 had the lowest rate of GRS, there is a slight increase in Quintile 5, however this is not significantly different (CI 0.9–2.9). Our results suggest a socioeconomic inequity in GRS, with those most disadvantaged more likely to experience serious harm from gambling. This reflects evidence from an earlier Victorian study that showed populations in disadvantaged areas are more likely to be exposed to high-intensity gambling and that losses in these areas are substantially higher than in less disadvantaged areas. In that study, 40% of the apparent effect of disadvantage was accounted for by the density of EGMs.^26^

Suicide prevention strategies in previous studies have primarily focused on the health sector: for example, training mental health professionals to screen individuals for gambling problems when they present with mental ill health or substance use issues; training primary health professionals to recognise and address potential suicide risk among people at high-risk of gambling harm; and incorporating gambling treatment expertise into mental health and drug and alcohol services.^21^, ^22^, ^23^^,^^27^^,^^28^ However, our finding that clinicians were aware of the deceased’s gambling in only 20% of cases suggests more effort is required to engage people who gamble with health services in the first place. Patients should be asked about gambling engagement and referred to appropriate support services. Referrals should seek to support immediate concerns, and support may be outside the clinical health setting, such as financial counselling and relationship support. There may also be a role for family, friends and colleagues, who were aware of the gambling in nearly three-quarters of cases, to support their loved ones to seek help. Wong et al. (2010) noted there is a need to enhance awareness of gambling harm within society more broadly and promote help-seeking behaviours among those at high-risk, including the development of outreach services that may help to enhance identification and help-seeking among this at-risk group.^23^

Two further important public health considerations arise in considering whether social networks might be a means to direct people who gamble at high-risk levels to clinical intervention and engagement with health services. The first consideration is the identification of 17 deaths of affected others in this study highlights the serious mental health impact that a person’s gambling can have on those who know them. There is evidently an urgent need to support people affected by another person's gambling. Any messaging encouraging them to seek help for a loved one’s gambling will need to be carefully designed so as not to convey responsibility for persuading the gambler to seek help.

The second consideration is that clinicians and health services should be prepared to treat patients for whom gambling harm is part of a more complex presentation. In most GRS cases in this study, there was evidence that the deceased experienced co-occurring stressors including mental illness, substance use, and relationship conflict. Gambling can cause or exacerbate a wide range of harms,^5^ including many of the stressors identified in this study; and can also potentially be a response to those stressors.

Coroners and other officials responsible for death investigations should consider auditing how they identify and gather information about GRS, and whether there are any opportunities for improvement. This study relied on the VSR to identify GRS. Suicide registers are a crucial source of data for suicide prevention efforts. Existing suicide registers should continue to be resourced, and in jurisdictions where they do not currently exist, they should be established. Having more information about GRS can assist in identifying opportunities for prevention. For example, one study found that EGMs have higher odds of being associated with suicidal events^20^; if the forms of gambling implicated in GRS are described in detail then this could have important implications for regulating the design, availability, and provision of products that carry a stronger association with suicide. This evidence can also assist in garnering support for measures outside the health system that can prevent suicide. Upstream universal interventions like binding pre-commitment systems and universal maximum limits on gambling losses are key preventive measures that will reduce gambling-related harms.

For many years the responsible gambling discourse has deflected attention from the negative effects of gambling, framing gambling as a recreational pursuit. This is damaging for those who experience harm; it creates stigma and shame as those harmed are perceived as flawed consumers.^29^ The gambling industry is now recognised as a key operator within the commercial determinants of health. Ultimately, stronger regulation of gambling should result in a reduction of GRS.

### Conclusions

Gambling is legal in many high-income countries and increasingly available in low- and middle-income countries.^12^ This study adds to the weight of evidence that gambling’s harms extend to suicidality and suicide.^15^, ^16^, ^17^ In this study we identified potential opportunities to prevent suicide and support people at risk of GRS, including drawing on their social networks to direct them to help and support; and to ensure health services are prepared and equipped to address gambling harm as part of a more complex health presentation rather than as a standalone issue. We also highlight the potential risk to people affected by another’s gambling, and the benefits of collecting more information about the context in which GRS occurs to inform the development of targeted interventions.

This study indicates that ineffective regulation of gambling can lead to devastating outcomes for people who gamble at high-risk levels, their families, and broader society. As argued by Pirkis et al. (2023) comprehensive responses to suicide prevention are needed, extending to suicide prevention in all policies.^30^ National suicide prevention strategies should recognise the social, and in particular commercial, determinants of gambling-related suicide. Intersecting areas include, for example, health, gambling, social services, communications, taxation and revenue, justice and coronial systems and beyond.

To systematically reduce GRS, rule-based regulatory measures grounded in public health principles are needed to prevent harm before it occurs. Appropriate measures include establishing centralised, universal account registration systems that operate across gambling operators and products. Such accounts should provide visibility of cumulative gambling losses and the capacity to set binding limits on losses (pre-commitment systems). Maximum universal limits on the amounts of money that can be lost on gambling products should also be introduced. Gambling advertising and promotions should be banned, as should features, and characteristics of products that encourage people to gamble for extended periods of time and/or chase gambling losses, such as losses disguised as wins, near misses and bonus bets.^31^ These interventions might be given further impetus for implementation and expansion if they were also framed as suicide prevention initiatives.

## Contributors

AR, JD and LB conceptualised the study. JD and CM established a coding interface for the gambling case database based on discussions with all authors. AR and JD undertook a literature review. AR and CM coded the cases. AR conducted statistical analysis and prepared the manuscript. HN advised on statistical analysis and reviewed AR's work. JD cross checked the coded cases. AR prepared the original draft of the manuscript. All authors reviewed the manuscript and provided input as the manuscript developed.

## Data sharing statement

The data contained in this study is sensitive and unable to be shared.

## Declaration of interests

AR is a Senior Research Fellow at Federation University, an Adjunct Senior Research Fellow at the Department of Forensic Medicine at Monash University, a Commissioner on the Lancet Public Health Commission on Gambling and member of WHO expert panel on gambling. AR has been an investigator on research funding from Suicide Prevention Australia Research Prevention Fund, Federation University, the Victorian Responsible Gambling Foundation (VRGF), Australia’s National Research Organisation for Women’s Safety and the Australian Commonwealth Department of Social Services. She has previously been employed by Deakin University, the Australian Institute of Family Studies and on grants funded by the Australian Research Council and VRGF. AR has received consultancy funding from WHO, Monash City Council and University of Waterloo, Ontario as well as travel funding from Federation University, Monash University, the Turkish Green Crescent Society, and the Winston Churchill Memorial Trust.

## Footnotes

f Australian Bureau of Statistics, Taxation Revenue, Australia https://www.abs.gov.au/AUSSTATS/abs@.nsf/DetailsPage/5506.02016-17?OpenDocument [last accessed 8 May 2023].

g Australian Bureau of Statistics. 310104 Population–states and territories, ABS: Canberra. Accessed 11 February 2022, from: https://www.abs.gov.au/statistics/people/population/national-state-and-territory-population/latest-release.

h Discover Victoria’s Diverse Population https://www.vic.gov.au/discover-victorias-diverse-population [last accessed 8 May 2023].

## References

[1] Hing N., O'Mullan C., Nuske E. (2020).

[2] Markham F., Doran B., Young M. (2016). The relationship between electronic gaming machine accessibility and police-recorded domestic violence: a spatio-temporal analysis of 654 postcodes in Victoria, Australia, 2005–2014. Soc Sci Med.

[3] Petry N.M., Weinstock J. (2007). Internet gambling is common in college students and associated with poor mental health. Am J Addict.

[4] Erickson L., Molina C.A., Ladd G.T., Pietrzak R.H., Petry N.M. (2005). Problem and pathological gambling are associated with poorer mental and physical health in older adults. Int J Geriatr Psychiatry.

[5] Langham E., Thorne H., Browne M., Donaldson P., Rose J., Rockloff M. (2015). Understanding gambling related harm: a proposed definition, conceptual framework, and taxonomy of harms. BMC Public Health.

[6] Browne M., Greer N., Armstrong T. (2017).

[7] Kourgiantakis T., Saint-Jacques M.-C., Tremblay J. (2013). Problem gambling and families: a systematic review. J Soc Work Pract Addict.

[8] Goodwin B.C., Browne M., Rockloff M., Rose J. (2017). A typical problem gambler affects six others. Int Gambl Stud.

[9] Browne M., Delfabbro P., Thorne H.B. (2023). Unambiguous evidence that over half of gambling problems in Australia are caused by electronic gambling machines: results from a large-scale composite population study. J Behav Addict.

[10] Gilmore A.B., Fabbri A., Baum F. (2023). Defining and conceptualising the commercial determinants of health. Lancet.

[11] Livingstone C., Rintoul A. (2020). Moving on from responsible gambling: a new discourse is needed to prevent and minimise harm from gambling. Public Health.

[12] Reynolds J., Kairouz S., Ilacqua S., French M. (2020). Responsible gambling: a scoping review. Crit Gambl Stud.

[13] van Schalkwyk M.C.I., Collin J., Eddleston M. (2023). Conceptualising the commercial determinants of suicide: broadening the lens on suicide and self-harm prevention. Lancet Psychiatry.

[14] Miller H.E., Thomas S.L., Smith K.M., Robinson P. (2016). Surveillance, responsibility and control: an analysis of government and industry discourses about “problem” and “responsible” gambling. Addict Res Theory.

[15] Marionneau V., Nikkinen J. (2022). Gambling-related suicides and suicidality: a review of qualitative evidence. Front Psychiatry.

[16] Andreeva M., Audette-Chapdelaine S., Brodeur M. (2022). Gambling-related completed suicides: a scoping review. Addict Res Theory.

[17] Karlsson A., Håkansson A. (2018). Gambling disorder, increased mortality, suicidality, and associated comorbidity: a longitudinal nationwide register study. J Behav Addict.

[18] Muggleton N., Parpart P., Newall P., Leake D., Gathergood J., Stewart N. (2021). The association between gambling and financial, social and health outcomes in big financial data. Nat Human Behav.

[19] Wardle H., Kesaite V., Tipping S., McManus S. (2023). Changes in the severity of problem gambling and subsequent suicide attempts: a longitudinal survey of young adults in Great Britain, 2018-20. Lancet Public Health.

[20] Bischof A., Meyer C., Bischof G. (2016). Type of gambling as an independent risk factor for suicidal events in pathological gamblers. Psychol Addict Behav.

[21] Markham F., Gobaud A.N., Mehranbod C.A., Morrison C.N. (2023). Casino accessibility and suicide: a county-level study of 50 US states, 2000 to 2016. Addiction.

[22] Blaszczynski A., Farrell E. (1998). A case series of 44 completed gambling-related suicides. J Gambl Stud.

[23] Wong P.W., Cheung D.Y., Conner K.R., Conwell Y., Yip P.S. (2010). Gambling and completed suicide in Hong Kong: a review of coroner court files. Prim Care Companion J Clin Psychiatry.

[24] Kaggwa M.M., Mamum M.A., Najjuka S.M. (2022). Gambling-related suicide in East African Community countries: evidence from press media reports. BMC Public Health.

[25] Gagnon K., Leclerc W. (2018). https://www.lapresse.ca/actualites/enquetes/201809/28/01-5198306-loterie-video-au-moins-400-suicides-lies-au-jeu-au-quebec.php.

[26] Rintoul A., Livingstone C., Mellor A., Jolley D. (2013). Modelling vulnerability to gambling-related harm: how disadvantage predicts gambling losses. Addict Res Theory.

[27] Gray H.M., Edson T.C., Nelson S.E., Grossman A.B., LaPlante D.A. (2021). Association between gambling and self-harm: a scoping review. Addict Res Theory.

[28] Séguin M., Boyer R., Lesage A. (2010). Suicide and gambling: psychopathology and treatment-seeking. Psychol Addict Behav.

[29] Rintoul A. (2023). Gambling, stigma, suicidality, and the internalization of the 'responsible gambling' mantra. Front Psychiatry.

[30] Pirkis J., Gunnell D., Hawton K. (2023). A public health, whole-of-government approach to national suicide prevention strategies. Crisis.

[31] Livingstone C., Rintoul A., de Lacy-Vawdon C. (2019).

