# Supplementary material for: Gambling-related suicide in Victoria, Australia: a population-based cross-sectional study
Source: Lancet Reg Health West Pac. 2023 Sep 12;41:100903. doi: 10.1016/j.lanwpc.2023.100903 (PMC10786645; doi:10.1016/j.lanwpc.2023.100903)
Supplement: Supplementary Table 1 [file mmc1.docx]

**Supplementary data**

The following table outlines the measures and categories of variables captured in this study.

**Table 1: Variables**

| **Data Item** | **Measure** | **Categories (if relevant)** |
| --- | --- | --- |
| **Gambling-Related Suicide** | | |
| Frequency of GRS | n (%) Male  n (%) Female  n (%) Total | Probable GRS  Possible GRS  Affected Other  Not GRS |
| Rate GRS | Average annual rate GRS / million Victorian adults | Probable GRS |
| **Sociodemographic characteristics** | | |
| Age | Mean Male  Mean Female  Mean Total  p value | - |
| Age Group (years) | n (%) Male  n (%) Female  n (%) Total | <18  18-24  25-34  35-44  45-54  55-64  65+ |
| Relationship status | n (%) Male  n (%) Female  n (%) Total | Not in a relationship  Married/ de facto/ domestic partner  Dating, other |
| Evidence born overseas | n (%) Male  n (%) Female  n (%) Total | Yes  No |
| Employment status | n (%) Male  n (%) Female  n (%) Total | Employed  Unemployed  Retired/Pensioner  Unable to work  Other* (prisoner, student, house duties)  Unknown |
| IRSD Quintile of usual residence | n (%) Total GRS  Total Rate GRS / 100,000 population  Total Rate Ratio (95% CI)  n (%) Victorian population / 100,000 | Quintile 1  Quintile 2  Quintile 3  Quintile 4 (reference)  Quintile 5  Unallocated/missing |
| Location of usual residence | n (%) Total GRS  Total Rate GRS / 100,000 population  Total Rate Ratio (95% CI)  n (%) Victorian population / 100,000 | Metropolitan  Non-metropolitan |
| **Gambling characteristics** | | |
| Evidence of a diagnosed gambling disorder | n (%) Male  n (%) Female  n (%) Total | Yes  No |
| Product(s) known to be most problematic (current or past) | n (%) Male  n (%) Female  n (%) Total | EGMs  Wagering (sport, animal racing both land-based and online)  Casino (product typically not specified) |
| Gambling known by | n (%) Male  n (%) Female  n (%) Total | Family/friends (partner, child, other family member, colleague)  Clinician (GP, mental health professional, other allied health professional) |
| Evidence of support for harmful gambling | n (%) Male  n (%) Female  n (%) Total | General Practitioner  Specialist gambling service Mental health professional  Other  Gamblers anonymous |
| Known stressors for person who gambles | n (%) Male  n (%) Female  n (%) Total | Relationship  Financial |
| Known impacts on person who gambles life | n (%) Male  n (%) Female  n (%) Total | Relationships (partner, child, family, friends, colleagues)  Job loss  Housing  Legal (civil and/or criminal) |

***** small numbers were collapsed to ‘other’ for deidentification purposes.
